# Effect of Vitamin D Supplementation on the Hemoglobin Level in Chronic Kidney Disease Patients on Hemodialysis: A Systematic Review and Meta-Analysis

**DOI:** 10.7759/cureus.40843

**Published:** 2023-06-23

**Authors:** Saad Ahmad, Hazrat Ullah, Moiz I Khan, Maryam Gul, Muhammad Saeed Ahmed, Maha Khalil, Mateen Ahmad, Abu baker Khan

**Affiliations:** 1 Orthopedic Surgery, Taj Medical Center, Nowshera, PAK; 2 Medicine, Khyber Medical College, Peshawar, PAK; 3 Accident and Emergency, Medical Teaching Institution (MTI) Divisional Headquarter (DHQ) Teaching Hospital, Dera Ismail Khan, PAK; 4 Internal Medicine, Taj Medical Center, Nowshera, PAK; 5 Orthopaedics, Mercy Teaching Hospital, Peshawar, PAK; 6 Gastroenterology and Hepatology, Shalamar Medical and Dental College, Lahore, PAK; 7 Surgery, Khyber Teaching Hospital, Peshawar, PAK; 8 Internal Medicine, Ayub Teaching Hospital, Abbottabad, PAK

**Keywords:** chronic kidney disease (ckd), anemia, hemodialysis, hemoglobin level, vitamin d supplementation

## Abstract

The objective of this study was to evaluate the impact of vitamin D supplementation on hemoglobin levels (Hb) in patients with chronic kidney disease (CKD) undergoing hemodialysis. A systematic search was conducted in electronic databases (PubMed/Medline, Cochrane Library, and Google Scholar) from inception to April 21, 2023. Inclusion criteria were applied to select relevant studies. Statistical analyses were performed using Review Manager 5.4.1. A random-effects model was used to address heterogeneity, and the mean difference (MD) with the corresponding 95% confidence interval (CI) was reported. Ten studies were included in the analysis, comprising seven clinical trials, two randomized clinical trials, and one retrospective observational study. Subgroup analysis was conducted based on the duration of follow-up: 12 weeks, three months, six months, 12 months, 15 months, and 18 months. A significant increase in hemoglobin levels was observed after 12 months (MD = -0.98 [95% CI -1.88, -0.08]; p = 0.03; I2 = 91%) and 18 months (MD = -1.80 [95% CI -2.56, -1.04]; p < 0.00001; I2 = Not applicable). However, there was no statistically significant relationship between vitamin D supplementation and hemoglobin levels at 12 weeks, three months, six months, and 15 months. The pooled analysis demonstrated a significant increase in hemoglobin levels with vitamin D supplementation (MD = -0.61 [95% CI -0.96, -0.26]; p = 0.03; I2 = 60.7%). This analysis highlights the significant role of vitamin D supplementation in improving anemia in patients with CKD undergoing hemodialysis. Vitamin D supplementation was found to significantly increase hemoglobin levels, particularly after 12 months and 18 months of supplementation.

## Introduction and background

Chronic kidney disease (CKD) is defined as having a glomerular filtration rate (GFR) of less than 60 mL/min per 1·73 m². It has an estimated global prevalence of >10% in the general population [1]. The most common etiology of CKD is diabetes and hypertension [2]. Hyperglycemia increases the filtration rate and renal blood flow. This leads to an increase in the GFR and may cause proteinuria. Elevated blood pressure can damage the kidneys by ischemic changes to the nephron, which ultimately leads to glomerular sclerosis, tubular atrophy, and interstitial nephritis [2]. It can be asymptomatic or can present with symptoms like edema, lethargy, pallor, cramps, bone pain, hematuria, cognitive impairment, and weight loss. Loss of albumin in the form of proteinuria, low erythropoietin levels with insufficient erythropoiesis, and inability to excrete toxins are the primary manifestation of CKD. Uremia can develop in CKD patients, and it can lead to dangerous complications like pericarditis, gastrointestinal bleeding, and encephalopathy [2]. 

Diagnosis can be made on the presence of symptoms, findings in urinary dipsticks, change in the GFR, and biopsy [3]. The goal of early treatment is to delay the progress to end-stage kidney disease. Patients need frequent blood transfusions for anemia. Patients are advised to decrease the consumption of eggs and red meat to prevent hyperphosphatemia. Statins and vitamin D supplements help in decreasing cholesterol and maintaining bone health. Exercise, fluid restriction, and diet help in controlling hypertension and edema. Metoclopramide and antihistamines provide relief from the symptoms of nausea and itching due to dry skin. End-stage kidney disease is established when the GFR is less than 15 mL/min per 1·73m². Renal replacement therapy which includes dialysis and kidney transplantation is the treatment for this stage [3]. 

Anemia and mineral bone disease are common complications of CKD [4]. Erythropoietin, produced by the kidney, drives the bone marrow to produce red blood cells and increase hemoglobin levels [5]. The kidney is responsible for converting vitamin D to calcitriol, and it regulates intestinal absorption of calcium and phosphate [6]. Vitamin D is also involved in the induction of erythropoiesis by increasing erythropoietin levels and stimulating erythrocyte precursor cell receptors [7]. 

 A randomized control trial conducted by Rianthavorn et al. has shown that vitamin D insufficiency leads to erythropoietin resistance in CKD and administration of vitamin D decreases the need for erythropoietin [8]. Another study found that vitamin D use in vitamin-D-deficient patients lessens the need for erythropoietin [9]. However, a meta-analysis conducted to see the relationship between vitamin D supplementation and hemoglobin concentration found no significant association between them [10]. 

The purpose of this systematic review and meta-analysis is to assess the relationship between vitamin D supplementation and hemoglobin levels in dialysis patients. Our review includes more recent articles that were not present in the previous meta-analysis. 

## Review

Method

Data Sources and Search Strategy

We used Preferred Reporting Items for Systematic Reviews and Meta-Analyses (PRISMA) to conduct our meta-analysis [11]. We searched PubMed/Medline, Cochrane Library, and Google Scholar from inception to 21st April 2023, using the search string: (vitamin D supplementation OR Calciferol OR cholecalciferol OR 1,25-Dihydroxycholecalciferol) AND (Anemia OR low Hb OR hemoglobin) AND (CKD OR chronic kidney disease OR chronic kidney failure OR dialysis OR hemodialysis). We also manually screened the articles.  

Eligibility Criteria

We used PICOS to formulate our eligibility criteria: P (Patients): chronic kidney disease in hemodialysis patients; I (Intervention): Vitamin D supplementation; C (Control): None; O (Outcome): Effect of Vitamin D supplementation on Hb levels; S (Studies): Observational studies and Randomized Controlled Trials.

Data Extraction and Quality Assessment of Studies 

Two impartial reviewers conducted computerized database searches. The studies were exported to the EndNote Reference Library version 20.0.1 (Clarivate Analytics) software, and duplicates were checked and eliminated. Two reviewers extracted data and assessed the quality of included studies concurrently and independently. 

The risk of biases from clinical trials was assessed, through the Risk Of Bias In Non-randomized Studies of Interventions (ROBINS-I): ROBINS-I approach, in seven domains: pre-intervention; bias due to confounding and bias in the selection of participants into the study; at intervention; bias in the classification of interventions, post-intervention; bias due to deviations from intended intervention, bias due to missing data, bias in measurement of outcomes, and bias in the selection of the reported result. The individual domains and overall risk-of-bias judgment were expressed on one of three levels: low risk of bias, unclear risk of bias, and high risk of bias. Based on these factors, the overall quality of evidence was deemed low, moderate, or high risk of bias (Table 1). 

**Table 1:** Details of Quality Assessment of Clinical Trials Robins-I

Study	Bias Due to Confounding	Bias in the Selection of Participants in the Study	Bias in the Classification of Interventions	Bias Due to Deviations from intended interventions	Bias Due to Missing Data	Bias in the Measurement of Outcomes	Bias in the Selection of the Reported Result	Net Risk of Bias
Saab et al. [12]	Unclear Risk	Low Risk	Low Risk	Low Risk	Low Risk	Low Risk	Unclear Risk	Low Risk
Albitar et al. [13]	Unclear Risk	Low Risk	Unclear Risk	Unclear risk	Low Risk	Low Risk	Unclear Risk	Moderate Risk
Manzoor et al. [14]	Unclear Risk	Low Risk	Unclear Risk	Low Risk	Low Risk	Low Risk	Unclear Risk	Low Risk
Neves et al. [15]	Unclear Risk	Low Risk	Low Risk	Low Risk	Low Risk	Low Risk	Unclear Risk	Low Risk
Goicoechea et al. [16]	Low Risk	Low Risk	Low Risk	Low Risk	Low Risk	Low Risk	Unclear risk	Low Risk
Agarwal et al. [17]	Low Risk	Low Risk	Low Risk	Low Risk	Low Risk	Low Risk	Low Risk	Low Risk
Firozjaei et al. [18]	Unclear Risk	Low Risk	Low Risk	Low Risk	Low Risk	Low Risk	Unclear risk	Low Risk

The risk of biases from randomized controlled trials was assessed through the Cochrane collaboration tool approach, in seven domains: adequate sequence generation, allocation concealment, blinding of participants and personnel, blinding of outcome assessment, incomplete outcome data, selective outcome reporting, and free of other bias (Table 2). 

**Table 2:** Details of Quality Assessment of Randomized Controlled Trials Using the Cochrane Collaboration Tool

Study	Adequate Sequence Generation	Allocation Concealment	Blinding of Participants and Personnel	Blinding of Outcome Assessment	Incomplete Outcome Data	Selective Outcome Reporting	Free of Other Bias	Net Risk of Bias
Obi et al. [19]	Low Risk	Low Risk	Low Risk	Low Risk	Low Risk	Unclear Risk	Low Risk	Low Risk
Ghasemian et al. [20]	Low Risk	Low Risk	Low Risk	Low Risk	Low Risk	Unclear Risk	Low risk	Low Risk

The Newcastle-Ottawa Scale (NOS) was used to grade the cohort studies' quality. NOS score 1-5 was considered a high risk for bias, 6-7 was moderate, and a score >7 was considered a low risk of bias (Table 3). 

**Table 3:** Quality Assessment of the Observational Study Using the Newcastle-Ottawa Scale

Studies	Selection (Maximum 4)				Comparability (Maximum 2)	Outcome (Maximum 3)			Total score
	Representativeness of the Exposed Cohort	Selection of the Non-Exposed Cohort	Ascertainment of Exposure	Demonstration That Outcome of Interest Was Not Present at Start of Study	Comparability of Cohorts on the Basis of the Design or Analysis	Assessment of Outcome	Was Follow-Up Long Enough for Outcomes to Occur	Adequacy of Follow Up of Cohorts	
Varim et al. [21]	1	1	1	1	2	1	1	1	9

Statistical Analysis

Review Manager (version 5.4.1; Copenhagen: The Nordic Cochrane Centre, The Cochrane Collaboration, 2020) was used for all statistical analyses. The data from studies were pooled using a random-effects model. Analysis of results was done by calculating the mean difference (MD) with respective 95% confidence interval (CI). The chi-square test was performed to assess any differences between the subgroups. Sensitivity analysis was performed to see if any individual study was driving the results and to implore reasons for high heterogeneity. As per Cumpston et al., the scale for heterogeneity was considered as follows: I2 = 25-60% - moderate; 50-90% - substantial; 75-100% - considerable heterogeneity, and p < 0.1 indicated significant heterogeneity [22]. A p < 0.05 was considered significant for all analyses. 

Results 

Search Strategy

**Figure 1:**
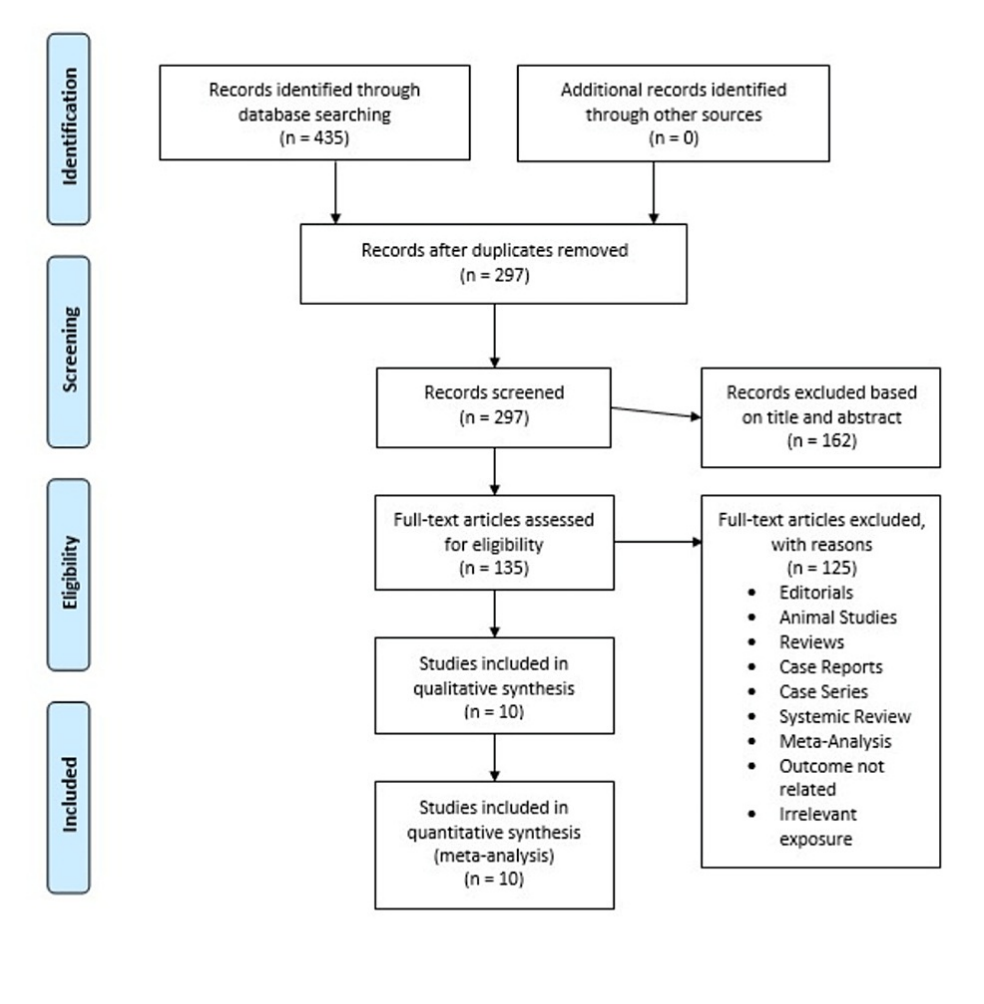
PRISMA Flow Diagram PRISMA: Preferred Reporting Items for Systematic Reviews and Meta-Analyses

Four hundred and thirty-three studies were retrieved from three search engines, 135 articles were selected for full text, and 10 studies were finalized for analysis [13-22]. Figure 1 shows the PRISMA flow chart. 

Baseline Characteristics

Table 4 shows the baseline characteristics of the selected studies [12-21]. A total of 878 patients were included in the analyses. The mean baseline Hb was 11.18 mg/dl and the mean age was 57.5 years. We had three studies whose intervention duration was three months, three studies with a duration of six months, three studies with a duration of 12 months, and one study with a duration of 18 months. Other details of dosage and baseline hemoglobin levels are provided in Table 4. 

**Table 4:** Baseline Characteristics N/A*= Not available; IU= International units

Study	Year	Duration	Total Patients	Mean Age (Years)	Dose of Vitamin D	Duration of Vitamin D Supplementation	Baseline Hemoglobin (mg/dl)	Net Risk of Bias
Albitar et al. [13]	1997	N/A*	12	59	6–7 mg per week	18 months	8.7	Moderate Risk
Goicoechea et al. [16]	1998	N/A*	28	55.5	2 ug i.v.	12 months	10.6	Low Risk
Varim et al. [21]	2016	N/A*	111	60.1	N/A*	6 months	11.1	Low Risk
Neves et al. [15]	2006	N/A*	11	73.6	2.33 mcg/pt/week	12 months	10.2	Low Risk
Saab et al. [12]	2007	May-Oct 2005	131	N/A*	50,000 IU monthly	6 months	11.8	Low Risk
Agarwal et al. [17]	2016	N/A*	186	57.6	50,000 IU/Week	12 months	11.1	Low Risk
Manzoor et al. [14]	2018	Jan-Apr 2017	210	50	0.04 μg/Kg 3 times a week	3 months	10.8	Low Risk
Obi et al. [19]	2020	Aug 2014- Sep 2015	96	- N/A*	3,000-IU/week, 9,000-IU/week	6 months	10.6	Low Risk
Firozjaei et al. [18]	2021	N/A*	38	48.3	50 000 IU/week, 1000 IU daily	3 months	8.9	Low Risk
Ghasemian et al. [20]	2022	N/A*	55	55.6	50,000 units/week	3 months	9.58	Low Risk

Publication Bias and Quality Assessment

**Figure 2:**
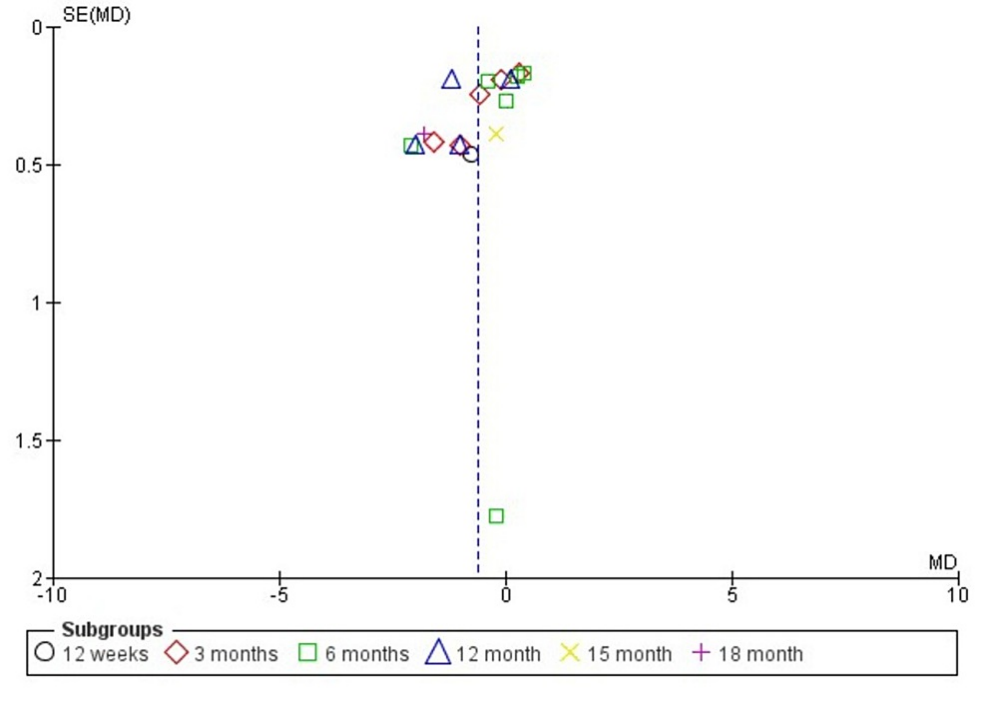
Funnel Plot Image credits: Abu baker Khan (Author)

The funnel plot (Figure 2) shows that there is a symmetry which indicates that there is no publication bias. 

Only one study showed a moderate risk of bias [13]. Others had a low risk of bias (Tables 1-3). 

Effect of Vitamin D Supplementation on Hemoglobin in Hemodialysis Patients With CKD

**Figure 3:**
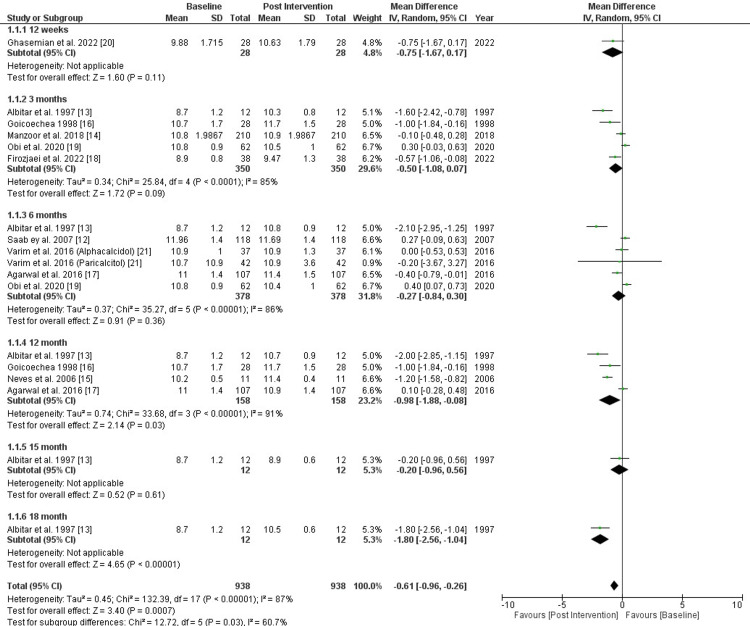
Forest Plot Showing the Effect of Vitamin D Supplementation on Hb Levels

Ten studies were assessed to evaluate whether vitamin D supplementation played a role in CKD patients undergoing hemodialysis [12-21]. Six subgroups were generated based on time for the follow-up: 12 weeks (one study), three months (five studies), six months (five studies), 12 months (four studies), 15 months (one study), and 18 months (one study). The forest plot (Figure 3) shows the quantitative analysis. There was a statistically significant increase in hemoglobin after 12 months (MD= -0.98 [95% CI -1.88, -0.08]; p= 0.03; I2= 91%) and 18 months (MD= -1.80 [95% CI -2.56, -1.04]; p< 0.00001; I2= Not applicable), while there was a statistically non-significant relationship between vitamin D supplements and hemoglobin levels after 12 weeks (MD= -0.75 [95% CI -1.67, 0.17]; p= 0.11; I2= Not applicable), three months (MD= -0.50 [95% CI -1.08, 0.07]; p= 0.09; I2= 85%), six months (MD= -0.27 [95% CI -0.84, 0.30]; p= 0.36; I2= 86%), and 15 months (MD= -0.20 [95% CI -0.96, -0.56]; p= 0.61; I2= Not applicable). Pooled results showed that vitamin D significantly increased hemoglobin in these patients (MD= -0.61 [95% CI -0.96, -0.26]; p= 0.03; I2= 60.7%) (Figure 3). 

Sensitivity Analysis 

One by one studies were removed to see their effect on the analysis but no study significantly showed an effect on the results. Thus, the results are robust. 

Discussion

In this systematic review and meta-analysis, we presented an assessment of evidence from 10 published studies to evaluate the role of vitamin D supplements in the improvement of anemia in CKD patients. The overall statistical analysis showed a significant role of vitamin D supplements in the improvement of anemia; however, the subgroup analysis suggested significant hemoglobin improvement only after 12 months and 18 months. The uncertainty in the subgroup analysis could be due to inconsistency in the available data. 

CKD is commonly associated with hypertension, diabetes, and glomerulonephritis [23]. Hypertension causes renal vasculature changes, leading to ischemia and immune responses that result in tubular atrophy and interstitial nephritis. Diabetes induces glomerular hyperfiltration, hyperglycemia, and cytokine production, causing glucose transport impairment and nephron damage [24]. Glomerulonephritis involves complement system activation, leading to endothelial damage and proteinuria [25]. CKD leads to sodium and fluid retention, edema, anemia, electrolyte imbalances, and osteodystrophy due to impaired phosphate excretion. These processes ultimately decrease the kidney's GFR, causing a range of symptoms and complications [26].

Complications of CKD including uremia and accumulation of other toxins can be detrimental to the patient. Uremia is defined as the buildup of urea in the blood, and it affects numerous functions of the body. Platelet dysfunction can lead to ecchymosis and gastrointestinal bleeding. Patients may complain of dry itchy skin and weight loss. It can cause pericarditis and encephalitis in patients which is especially worrisome in patients. 

The literature showed a debate on the management of CKD patients; the guidance combined the management for the general population, general patients’ management, and CKD-specific guidelines [27]. Therefore, it requires detailed knowledge of the recommendations on vitamin D. Christodoulou et al. conducted a meta-analysis to assess the impact of vitamin D supplements on CKD patients. The 22 trials based on statistical analysis showed that vitamin D supplements have a nonsignificant effect on parathyroid hormone. They reported that the effect of vitamin D supplements in CKD patients was inconsistent; however, calcifediol and its analogs suppressed PTH [27]. 

The association of vitamin D with anemia is not scanty in the literature; a meta-analysis conducted by Liu et al. reported that vitamin D deficiency increases the risk of anemia [28]. Sooragonda et al. tested the effectiveness of high-dose vitamin D supplements in iron deficiency anemia patients. The author reported non-significant improvement in hemoglobin levels in comparison to the control group [29]. However, the review pooling the results from the trials showed inconclusive results to demonstrate the significance of vitamin D supplements on anemia [30]. A systematic review and meta-analysis concluded that supplementary vitamin D has no significant effect on hemoglobin or ferritin levels; however, it has significant effects on transferrin saturation and iron status [10]. Several trials report the effect of vitamin D supplements to improve anemia in CKD patients. However, we were unable to find any meta-analysis or systematic review that observed the effects of vitamin D supplements. It is postulated that the hepcidin-lowering effect of vitamin D and its immunomodulatory property contribute to the lowering of erythropoietin-stimulating agents. Activated vitamin D receptor is also considered a significant contributor to stimulating the growth of erythroid progenitors, resulting in iron consumption, therefore, lowering the hepcidin levels. 

Our statistical results support our hypothesis that vitamin D supplements can improve hemoglobin concentrations. However, the results are inconsistent. The in-group high heterogeneity and heterogeneity among the subgroups are also a drawback to the reliability of our results. Among the included studies, only Obi et al. reported non-significant improvement in the hemoglobin levels, and supplementation of cholecalciferol does not decrease the serum hepcidin level [19]. The author reported that the hepcidin was lower than the control group; however, it might not be directly related to vitamin D supplementations, they explain that at the sixth month, the effect of supplementations was offset due to a reduction in dose or could be due to non-effectiveness of erythropoietin-stimulating agents on the hepcidin level [19]. In contrast, all the included studies favor vitamin D supplements for the improvement of anemia. Albitar et al. reported the longest follow-up, the study reported statistically significant improvement of anemia in three months, six months, 12 months, and 18 months with alfacalcidol supplements [13]. 

Manzoor et al. provided the largest patient data of 210 patients on this subject. The observational study showed that paricalcitrol therapy was not significant to improve the hemoglobin levels and erythropoietin-stimulating agents, despite the agent notably decreasing the C-reactive protein levels to a potential amount to improve anemia management [14]. A blinded trial conducted by Ghasemian et al. used oral calcium-D tablets and reported a significant increase in hemoglobin level, more than 1 unit, in comparison to the control group. However, there is no statistically significant improvement when comparing baseline and endpoint hemoglobin. The study was also limited by the duration of the follow-up, only data for twelve weeks were reported [20]. 

Despite the pooled data, our study showed statistically significant improvement in hemoglobin levels with the vitamin D supplements, and the novelty of this study to open the door for further research on this topic is a major strength of our study, but the variability in the vitamin D agents used and their dosage can be a drawback to study. This could be a significant reason for the high ingroup heterogeneity. The neglection toward nutritional intake and prescribed agents to the included population can also be a major contributor to statistical results along or instead of supplementary vitamin D. We were able to compare the results with a control group due to data unavailability, we believe that larger blinded trials can be a breakthrough in this subject. 

Limitations 

Our study is limited due to the following reasons: (a) fewer patients were included in our analysis; (b) different vitamin D supplementations were pooled together; (c) stratification based on dosage was not conducted. However, these factors were pivotal in conducting this study. 

## Conclusions

Our meta-analysis yielded notable findings regarding the effects of vitamin D supplementation on hemoglobin levels in patients with CKD. The results demonstrated statistically significant improvements, particularly observed after a duration of 12 months. However, it is recommended that larger-scale trials be conducted to gain deeper insights into this subject matter and enhance our understanding.
